# Evaluation of the BioFire^®^ FilmArray^®^ Pneumonia Panel with Conventional Bacterial Culture in Conjunction with Leukocyte Esterase Test

**DOI:** 10.3390/diagnostics13111847

**Published:** 2023-05-25

**Authors:** In Young Yoo, Hyun Soo Seok, Joo An Kwon, Jongmin Lee, Sungjin Jo, Soo Young Kim, Yeon-Joon Park

**Affiliations:** 1 Department of Laboratory Medicine, Seoul St. Mary’s Hospital, College of Medicine, The Catholic University of Korea, Seoul 06591, Republic of Korea; yiy00@naver.com (I.Y.Y.); 98030618@cmcnu.or.kr (H.S.S.);; 2 Division of Pulmonary and Critical Care Medicine, Department of Internal Medicine, Seoul St. Mary’s Hospital, College of Medicine, The Catholic University of Korea, Seoul 06591, Republic of Korea; 3 Department of Laboratory, Eunpyeong St. Mary’s Hospital, College of Medicine, The Catholic University of Korea, Seoul 03382, Republic of Korea; 4 Infectious Disease Laboratory Research Center, Eunpyeong St. Mary’s Hospital, College of Medicine, The Catholic University of Korea, Seoul 03382, Republic of Korea; 5 Department of Laboratory Medicine, St. Vincent’s Hospital, College of Medicine, The Catholic University of Korea, Suwon 16247, Republic of Korea

**Keywords:** BioFire^®^ FilmArray^®^ pneumonia panel, community-acquired pneumonia, conventional culture, bacterial burden, leukocyte esterase, sputum quality

## Abstract

We evaluated the performance of the BioFire^®^ FilmArray^®^ Pneumonia panel (PN-panel) in detecting bacterial pathogens by comparing it to cultures and to the usefulness of the leukocyte esterase (LE) urine strip test. Between January and June 2022, a total of 67 sputum specimens were obtained from community-acquired pneumonia patients. The PN-panel and LE test were performed simultaneously with conventional cultures. The pathogen detection rates of the PN-panel and culture were 40/67 (59.7%) and 25/67 (37.3%), respectively. The concordance rate between the PN-panel and culture was high (76.9%) when the bacterial burden was high (10^7^ copies/mL), but it was low (8.6%) when it was 10^4−6^ copies/mL, irrespective of the sputum quality. According to the LE positivity, the overall culture positive rate and PN-panel positive rate were significantly higher among the LE-positive specimens (23/45, 31/45) than among the LE-negative specimens (2/21, 8/21). Moreover, the difference in concordance rate between the PN-panel test and culture was significant according to the LE positivity, but not the Gram stain grading. In conclusion, the PN-panel showed high concordance when the bacterial burden was high (10^7^ copies/mL) and ancillary use of LE test will be helpful in interpreting the PN-panel results, especially when the copy number of bacterial pathogens is low.

## 1. Introduction

Community-acquired pneumonia (CAP) is associated with high mortality and morbidity, particularly among adults >65 years of age [1]. The most recent American Thoracic Society (ATS)/Infectious Diseases Society of America (IDSA) CAP 2019 guidelines recommended a pretreatment Gram stain and culture for severe CAP and required admission to the hospital [2]. The diagnostic utility of Gram stains and cultures for respiratory specimens has been debated for several years due to the poor yield of Gram stains and cultures [3]. Jain et al. demonstrated that pathogens were detected only in 38% (853/2259) of the CAP patients requiring hospitalization [4]. Factors influencing the low yield of conventional cultures include (1) sputum quality [5], (2) fastidious growth requirements (such as *Streptococcus pneumoniae* and *Hemophilus influenzae*), and (3) exposure to antibiotics prior to culture [6]. In addition, on average, 30% of sputum specimens submitted to the laboratory are rejected based on Gram stain characteristics [7].

To overcome these limitations, several multiplex PCR-based tests for detecting bacterial/viral pathogens and antibiotic resistance markers have been developed. The BioFire^®^ FilmArray^®^ Pneumonia panel (PN-panel; BioFire Diagnostics LLC, Salt Lake City, UT, USA) is a multiplexed nested PCR assay which can detect fifteen common bacterial targets, three atypical pneumonia targets, eight common respiratory viruses, and seven antibiotic resistance markers in approximately 1 h. To assist differentiation between true infection and simple colonization, this assay provides semi-quantitative results for fifteen bacterial pathogens [8], but it does not determine the white blood cell count. In addition, there is no comment on the sputum quality in the CLSI guidelines for molecular methods [9]. 

The rapid urine strip test was originally designed as a semi-quantitative test for the presence of neutrophils in urine through the detection of leukocyte esterase (LE) enzyme activity. However, in BAL fluid, a correlation between LE level as detected by the reagent strip and the presence of neutrophils was shown [10], and it was shown to distinguish between exudates and transudates in pleural effusions [11].

Therefore, in this study, we aimed to compare the PN-panel to the conventional culture for the detection of bacterial pathogens and compared the rate of PN positivity according to the Gram stain and LE results of the sputum specimens.

## 2. Materials and Methods

### 2.1. Study Population 

The sputum specimens were collected from patients diagnosed as having severe CAP based on the IDSA/ATS consensus guidelines. Between January 2022 and June 2022, a total of 67 sputum specimens were obtained from CAP patients within 48 h after hospital admission. When the sputum specimens were received in the microbiology laboratory, the PN panel was performed simultaneously to the conventional culture. Remaining specimens were frozen at −70 °C until they were evaluated for discrepant analysis. 

### 2.2. Conventional Culture

All of the sputum specimens were processed using standard laboratory procedures [12]. Briefly, the Gram stain was performed after vortexing and if necessary, the purulent sputum specimen was homogenized with sputazyme (Kyoguto, Tokyo, Japan). The specimens were graded according to the Murray and Washington grading system [13]; sputum specimens showing grades of 4, 5, or 6 were considered as adequate specimens for culture [14]. However, in this study, considering that opportunistic pathogens can cause infections when microbial homeostasis is disrupted [15], all of the sputum specimens were inoculated onto 5% sheep blood agar, CHOC-VBC agar [16], and MacConkey agar plates and incubated at 35 °C in 5% CO_2_ for 48 h, regardless of the quality of sputum; we remarked the predominant growth of a single pathogen and considered the results as culture-positive.

All of the pathogenic bacteria were identified using matrix-assisted laser desorption ionization time-of-flight mass spectrometry with an ASTA MicroIDSys system (ASTA, Suwon, Republic of Korea) and/or VITEK-MS (bioMérieux, Marcy-l’Etoile, France).

### 2.3. BioFire FilmArray Pneumonia Panel (PN-Panel)

According to the manufacturer’s instructions, a PN-panel was performed by using a flocked swab (provided) from submitted sputum specimens before the sputazyme treatment. A swab sample was mixed with a sample buffer and injected into the PN-panel pouch. Then, the reagent pouch was inserted into the FilmArray instrument for analysis. For 15 bacterial pathogens, semi-quantitative results were reported as bins (i.e., 10^4^, 10^5^, 10^6^, and ≥10^7^ copies/mL). Three residual atypical bacteria, eight respiratory viruses, and seven antibiotic resistance markers were reported as “detected” or “not detected”. Hands-on time was less than 5 min, and the total analysis time was around 1 h.

### 2.4. Leukocyte Esterase (LE) Strip Test

We used the Multistix 10 SG reagent strip (Siemens Healthcare Diagnostics Inc., Munich, Germany) to evaluate the usefulness of the LE test in comparison with sputum grading by Gram stain. [17,18]. The strip was dipped into the sputum specimens and read after 2 min according to the manufacturer’s instruction. Based on the color change, the results were recorded as negative, 1+, 2+, and 3+. The investigator who interpreted the culture results was blinded to the LE test results. 

### 2.5. Discrepancy Analysis for Bacterial Pathogens

Discrepancy analysis was performed for specimens that showed positive PN-panel results and negative conventional culture results. For conventional PCR, DNA was extracted from 200 μL of frozen specimens using the QIAamp DNA mini kit (Qiagen, Hilden, Germany). The applied PCR primers for each target are described in Table S1 [19,20,21]. 

### 2.6. Statistical Analysis 

The correlation between the PN-panel and the conventional culture was investigated in the form of a positive percent agreement (PPA), a negative percent agreement (NPA), and an overall percent agreement (OPA) at 95% confidence intervals (95% CI). Chi-square or Fisher’s exact test were used to assess group differences in categorical variables. A *p*-value < 0.05 was considered statistically significant. All statistical analyses were performed using the SPSS software (version 24.0; SPSS Inc., Chicago, IL, USA).

## 3. Results

### 3.1. Pathogens Detected by PN-Panel vs. Conventional Culture

In comparison to the conventional culture, the PN-panel exhibited a substantially higher pathogen detection rate of 59.7% compared to 37.3%, with a significant *p*-value of 0.01 (Table 1). In 28 specimens, the PN-panel detected one bacterial target, and an identical pathogen was detected by cultures in 13 of those specimens. In one specimen, *P. aeruginosa* (10^6^ copies/mL) was detected in the PN-panel and *Corynebacterium striatum* was detected in the conventional culture. In eight out of twelve samples in which more than two bacteria were detected in the PN-panel, one or more of the same bacteria were cultured. Three specimens showed PN-panel-negative but culture-positive results; they were off-target pathogens for the PN-panel: *C. striatum* (*n* = 2) and *Burkholderia gladioli* (*n* = 1).

The OPA was calculated with respect to the conventional culture (Table 2). Among the 14 bacterial targets included in the PN-panel, for eight pathogens (*Acinetobacter baumannii*, *Escherichia coli*, *H. influenzae*, *Klebsiella pneumoniae*, *P. aeruginosa, S. aureus*, *Streptococcus agalactiae*, and *S. pneumonia*), PPA was 100%. For the remaining six pathogens, a PPA could not be calculated because no positive result was obtained with the cultures. The NPA was >95% for ten pathogens, but the four pathogens demonstrating a NPA of <95.0% were *H. influenzae* (92.4%), *K. pneumoniae* (90.5%), *P. aeruginosa* (90.2%), and *S. aureus* (89.6%). 

According to the quantity of pathogens detected in the PN-panel, the concordance rate was high (20/26, 76.9%) when the bacterial burden was ≥10^7^ copies /mL, but it was very low (3/35, 8.6%) when the bacterial burden was 10^4−6^ copies/mL, irrespective of the sputum quality as determined by Gram stains. Moreover, among the 26 isolates detected from inadequate specimens, in as many as 10 isolates, both the PN-panel and culture were positive and the bacterial burden was high (Table 3). 

**Figure 1:**
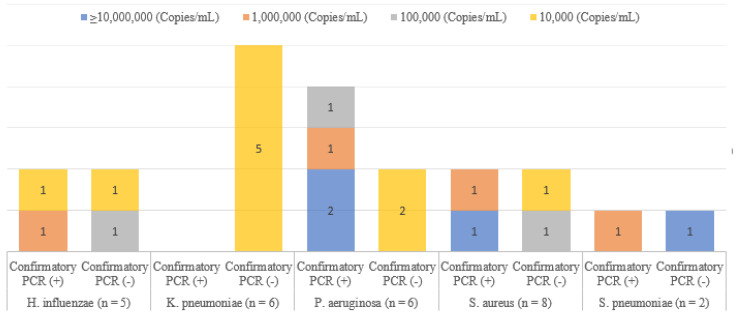
Summary of discrepancy analyses by confirmatory PCR in five representative analytes. (For some results, discrepant analysis could not be performed due to insufficient residual sample volume: *H. influenzae* (*n* = 1), *K. pneumoniae* (*n* = 1), and *S. aureus* (*n* = 4)).

Among these 27 discordant isolates, we performed an additional discordant analysis with conventional PCR (Figure 1) for the 21 cases which were available. As a result, nine isolates were identified in confirmatory PCR, and twelve were not detected. While the bacterial burden of all but two of the nine isolates was 10^6^ or ≥10^7^ copies/mL, the bacterial burden of all but one of the twelve isolates not detected in conventional PCR was 10^4^ or 10^5^ copies/mL.

### 3.2. Correlation between LE Test and Sputum Quality Grading, Culture-Positive Rate, and PN-Panel-Positive Rate

The LE strip tests were performed for all but one specimen, which was too mucoid. Looking into the correlation between the sputum gradings by Gram stain and the LE test results, the proportion of LE positivity was significantly higher (31/39, 79.5%) among the adequate specimens than among the inadequate specimens (14/28, 50.0%) (*p* value 0.02), indicating a good correlation between the gradings by Gram stain and the LE tests (Table 4). 

According to the LE positivity, the positivity rate of the PN-panel was significantly higher among the LE-positive specimens (31/45, 68.9%) than among the LE-negative specimens (8/21, 38.1%) (*p* value 0.03) (Table 4). Additionally, the overall culture-positive rate was significantly higher among the LE-positive (23/45, 51.1%) than among the LE-negative specimens (2/21, 9.5%) *(p* value 0.001) and even among the 28 inadequate sputum samples, while only one out of thirteen LE-negative samples were culture-positive, and more than half (nine out of fourteen) of the LE-positive samples were culture-positive (7.7% vs. 64.3%, *p* value 0.004) (Table S2).

## 4. Discussion

The pathogen detection rate using the PN-panel was significantly higher than the rate using conventional cultures (59.7% vs. 37.3%, *p* value 0.01). All bacteria (23/23, 100%) detected by conventional cultures were also identified by PN-panels, and 39 additional bacteria were identified by PN-panels alone. The concordance rate was high (20/26) when the bacterial load was high (10^7^ copies/mL), but it was much lower (3/35) when the bacterial load was 10^4−6^ copies/mL, which is in line with a previous study [8]. This might be due to the reporting guidelines for a respiratory culture, which suggest that potential upper respiratory normal flora should be reported when present at high quantities in sputum and when representing predominant growth [7]. Rouby et al. set the threshold for positivity on cultures as 10^7^ CFU/mL for sputum specimens, 10^4^ CFU/mL for BAL, and 10^5^ CFU/mL for endotracheal aspirates [22]. 

Considering that many members of the human respiratory tract microbiota are colonizing opportunistic pathogens that are normally harmless, but that can cause infections when the microbial homeostasis becomes disrupted [15], and a recent study in which it was revealed that the risk of exacerbation of bronchiectasis and long-term outcomes is associated with the reduced diversity of the sputum microbiome, particularly when dominated by the bacterial genera *Pseudomonas*, *Enterobacteriaceae*, and *Stenotrophomonas* [23], the bacterial copy number reported by the PN-panel should be taken into account when interpreting the PN-panel results. However, the PN-panel still has limitations in that it does not reflect the quality of the specimens. Based on our results, the LE test showed a good correlation with sputum grading by Gram stain. Furthermore, the overall culture positive rate, PN-panel positive rate, and concordance rate were significantly higher among the LE-positive specimens than the LE-negative specimens. Taken together, this finding suggests that the LE test can be used as a complement to sputum grading by Gram stain when interpreting PN-panel results or for conventional cultures.

In addition, we found that among the inadequate specimens, when the specimens were LE-positive, more than half of them showed the predominant growth of a single pathogen. Considering that the urine dipstick test for LE showed higher sensitivity than a sputum smear microcopy for leukocyte examination [17], these findings suggest that the LE test can be used as an ancillary test for the determination of sputum quality to reduce the specimens rejected for culture and can be used a marker of infection vs. colonization. Further studies with greater numbers of specimens are needed. 

It is also noteworthy that a *Legionella* sp. was unexpectedly detected by a PN-panel and was confirmed by PCR and sequencing. Considering that conventional cultures for *Legionella* spp. are not routinely performed and that the sensitivity is much lower than PCR (50% vs. 92%), use of the PN-panel will be helpful for detecting *Legionella* spp. [24]. 

In conclusion, the PN-panel showed high concordance when the bacterial burden was high (10^7^ copies/mL), and ancillary use of the LE test was helpful in interpreting the PN-panel results, especially when the copy number of bacterial pathogens was low, and in increasing the cultures’ sensitivity by reducing the number of inadequate specimens that were not proceeded for culture.

## Figures and Tables

**Table 1:** Number of sputum samples in which bacterial pathogens were detected or not detected using the FilmArray Pneumonia panel and conventional cultures.

FilmArray Pneumonia Panel (Number of Detected Pathogens)	Conventional Culture	
	Detected	Not Detected
Not detected (*n* = 27)	3 ^1^	24
1 (*n* = 28)	14 ^2^	14
2 (*n* = 5)	2	3
3 (*n* = 4)	3	1
4 (*n* = 3)	3	0
Total (*n* = 67)	25	42

^1^  *C. striatum* (*n* = 2), *B. gladioli* (*n* = 1). ^2^ Thirteen out of fourteen samples showed the same results in both the PN-panel and conventional culture, but one sample showed different results, being *P. aeruginosa*-positive in the PN-panel and *C. striatum*-positive in the conventional culture.

**Table 2:** Comparison of FilmArray Pneumonia panels and conventional cultures for each FilmArray Pneumonia panel bacterial target.

FilmArray Pneumonia Panel	Number of Results for FilmArray Pneumonia Panel/Conventional Culture				OPA (95% CI)
	(+/+)	(+/−)	(−/+)	(−/−)	
*A. baumannii*	2	3	0	62	95.5 (87.5–99.0)
*E. cloacae* complex	0	1	0	66	98.5 (92.0–99.9)
*E. coli*	2	2	0	63	97.0 (89.6–99.6)
*H. influenzae*	1	5	0	61	92.5 (83.4–97.5)
*K. aerogenes*	0	1	0	66	98.5 (92.0–99.9)
*K. oxytoca*	0	1	0	66	98.5 (92.0–99.9)
*K. pneumoniae*	4	6	0	57	91.0 (81.5–96.6)
*M. catarrhalis*	0	1	0	66	98.5 (92.0–99.9)
*P. aeruginosa*	6	6	0	55	91.0 (81.5–96.6)
*S. marcescens*	0	1	0	66	98.5 (92.0–99.9)
*S. aureus*	6	8	0	53	88.1 (77.8–94.7)
*S. agalactiae*	1	1	0	65	98.5 (92.0–99.9)
*S. pneumoniae*	1	2	0	64	97.0 (89.6–99.6)
*L. pneumophila*	0	1	0	66	98.5 (92.0–99.9)
Total	23	39	0	876	95.8 (94.4–97.0)

OPA, overall percent agreement; CI, confidence interval.

**Table 3:** Concordance between FilmArray Pneumonia panels and conventional cultures according to the bacterial burden and sputum quality.

PN-Panel Result (Copies/mL)	Sputum Quality				Concordance Rate
	Adequate		Inadequate		
	PN-Panel (+)/ Conventional Culture (+)	PN-Panel (+)/ Conventional Culture (−)	PN-Panel (+)/ Conventional Culture (+)	PN-Panel (+)/ Conventional Culture (−)	
10^4^ (*n* = 17)	1	10	0	6	1/17
10^5^ (*n* = 8)	0	4	0	4	0/8
10^6^ (*n* = 10)	1	4	1	4	2/10
≥10^7^ (*n* = 26)	11	4	9	2	20/26
Total	13	22	10 ^1^	16	23/61

PN-panel, FilmArray Pneumonia panel. ^1^ For inadequate specimens, 10 bacterial isolates (*K. pneumoniae* (*n* = 4), *P. aeruginosa* (*n* = 2), *S. aureus* (*n* = 2), *E. coli* (*n* = 1), and *S. pneumoniae* (*n* = 1)) were confirmed to have been cultured in a significant amount.

**Table 4:** FilmArray Pneumonia panel results according to the leukocyte esterase grades and sputum quality.

Urine Strip Grade for Leukocyte Esterase	Sputum Quality (Adequate) (*n* = 39)		Sputum Quality (Inadequate) (*n* = 28)	
	PN-Panel (+)	PN-Panel (−)	PN-Panel (+)	PN-Panel (−)
Negative (*n* = 21)	4	4	4	9
Positive (1+, 2+, 3+) (*n* = 45)	20	11	11	3
Mucoid (*n* = 1)	0	0	1	0
Total	24	15	16	12

PN-panel, FilmArray Pneumonia panel.

## Data Availability

The data presented in this study are available upon request from the corresponding author.

## Supplementary Materials

The following supporting information can be downloaded at https://www.mdpi.com/article/10.3390/diagnostics13111847/s1: Table S1: Primer information for conventional PCR; Table S2: Conventional culture results according to the leukocyte esterase grade and sputum quality.
