# Supplementary material for: Evaluation of the BioFire® FilmArray® Pneumonia Panel with Conventional Bacterial Culture in Conjunction with Leukocyte Esterase Test
Source: Diagnostics (Basel). 2023 May 25;13(11):1847. doi: 10.3390/diagnostics13111847 (PMC10252269; doi:10.3390/diagnostics13111847)
Supplement: Supplementary file 1 [file diagnostics-13-01847-s001.zip › diagnostics-2345264-supplementary.pdf]

Table S1. Primer information for conventional PCR

Targets	Sequence of primer (5′ → 3′)	PCR product size	References
K. pneumoniae	ATT TCT CCG GCG TCA AGT GT<br>CTC AAC ATC GTC GCA AAG GC	202	[19]
S. aureus	CGC AAA CTG TTG GCC ACT AT<br>CTC GCC ATC ATG ATT CAA GT	293	
S. pneumoniae	TTG ACC CAT CAG GGA GAA AG<br>CTT GAT GCC ACT TAG CCA AC	349	
P. aeruginosa	GAT GGA AAT GCT GAA ATT CG<br>GGA CGC TCT TTA CCA TAG GA	444	
H. influenzae	GCG AAA GTC CAA GCC TCT CT<br>TCA CCG TAA GAT ACT GTG CCT	582	
Legionella spp.	GGCGACCTGGCTTC<br>GGTCATCGTTTGCATTTATATTTA	101	[20]
Legionella pneumophilia	GGCCAATAGGTCCGCCAACG<br>GGTGACTGCGGCTGTTATGG	632	[21]

Table S2. Conventional culture results according to the leukocyte esterase grade and sputum quality.

Urine strip grade for leukocyte esterase	Sputum quality (adequate) (n = 39)		Sputum quality (inadequate) (n = 28)	
	Conventional culture (+)	Conventional culture (-)	Conventional culture (+)	Conventional culture (-)
Negative (n = 21)	1	7	1	12
Positive (1+, 2+, 3+) (n = 45)	14	17	9	5
Mucoid (n = 1)	0	0	0	1
Total	15	24	10	18
